# Development of novel thoracic retractor for resuscitative thoracotomy

**DOI:** 10.1186/s13049-025-01423-1

**Published:** 2025-06-17

**Authors:** Shoichiro Urabe, Junya Shimazaki, Tomohisa Izutani, Tsuyoshi Hata, Mamoru Uemura, Hidetoshi Eguchi, Yuichiro Doki, Kiyokazu Nakajima

**Affiliations:** 1 Department of Next Generation Endoscopic Intervention (Project ENGINE), Graduate School of Medicine, Osaka University, Suite 0802, BioSystems Bldg., 1-3, Yamadaoka, Suita, 565-0871 Osaka Japan; 2 Department of Gastroenterological Surgery, Graduate School of Medicine, Osaka University, 2-2, E-2, Yamadaoka, Suita, 565-0871 Osaka Japan; 3 Department of Emergency and Critical Care Medicine, Kansai Medical University, 2-3-1 Shinmachi, Hirakata, Japan; 4 Kajitech Medical Co., Ltd, 3-8-43, Shinomiya, Kadoma, 571-0017 Osaka Japan

**Keywords:** Thoracic retractor, Rib retractor, Rib spreader, Resuscitative thoracotomy, Resuscitation, Thoracotomy

## Abstract

**Background:**

Resuscitative thoracotomy (RT) is a critical intervention for patients in traumatic cardiac arrest or hemorrhagic shock, where survival is highly dependent on the time required to perform the procedure. Despite its urgency, RT is still conducted using traditional thoracic retractors originally designed for scheduled surgeries, which pose challenges in emergency settings. To address these limitations, we developed a novel thoracic retractor optimized for RT and evaluated its performance compared to a conventional model.

**Methods:**

The novel retractor was designed with an arrow-shaped hook for improved intercostal insertion and a continuously rotatable handle to enhance procedural efficiency. A comparative study using excised porcine thoraxes was conducted to assess its performance. Six cm incisions were made in the intercostal spaces bilaterally before retractor insertion. Evaluators inserted the device, performed three handle rotations, and repeated the procedure using the other retractor on the contralateral side. The primary outcome was the time required for three rotations, while secondary outcomes included ease of insertion, ease of rotation, and hook stability, rated on a 6-point scale by evaluators.

**Results:**

Ten surgeons (*n* = 10) performed thoracotomy using both the novel and conventional retractors. Comparison of the time required for three handle rotations between the novel and conventional retractors demonstrated a statistically significant reduction with the novel retractor. The median time to complete three rotations was 16.0 [11.7–19.1] seconds with the novel retractor, compared to 7.0 [5.3–8.5] seconds with the conventional model (*P* < 0.01). The ease of insertion was rated significantly higher with the novel retractor compared to the conventional model (6.0 [5.5–6.0] vs. 2.5 [2.0–3.0], *P* < 0.01). The ease of rotation was also rated significantly higher with the novel retractor than with the conventional model (5.5 [5.0–6.0] vs. 2.5 [1.0–3.5], *P* < 0.01). In the evaluation of the hook stability, no significant difference was observed between the novel and conventional retractors (*P* = 1.0).

**Conclusions:**

The novel thoracic retractor enables faster and easier thoracotomy compared to conventional model. Given the strong association between time and RT prognosis, this device is well-suited for RT procedures requiring rapid execution.

**Supplementary Information:**

The online version contains supplementary material available at 10.1186/s13049-025-01423-1.

## Background

**Fig. 1:**
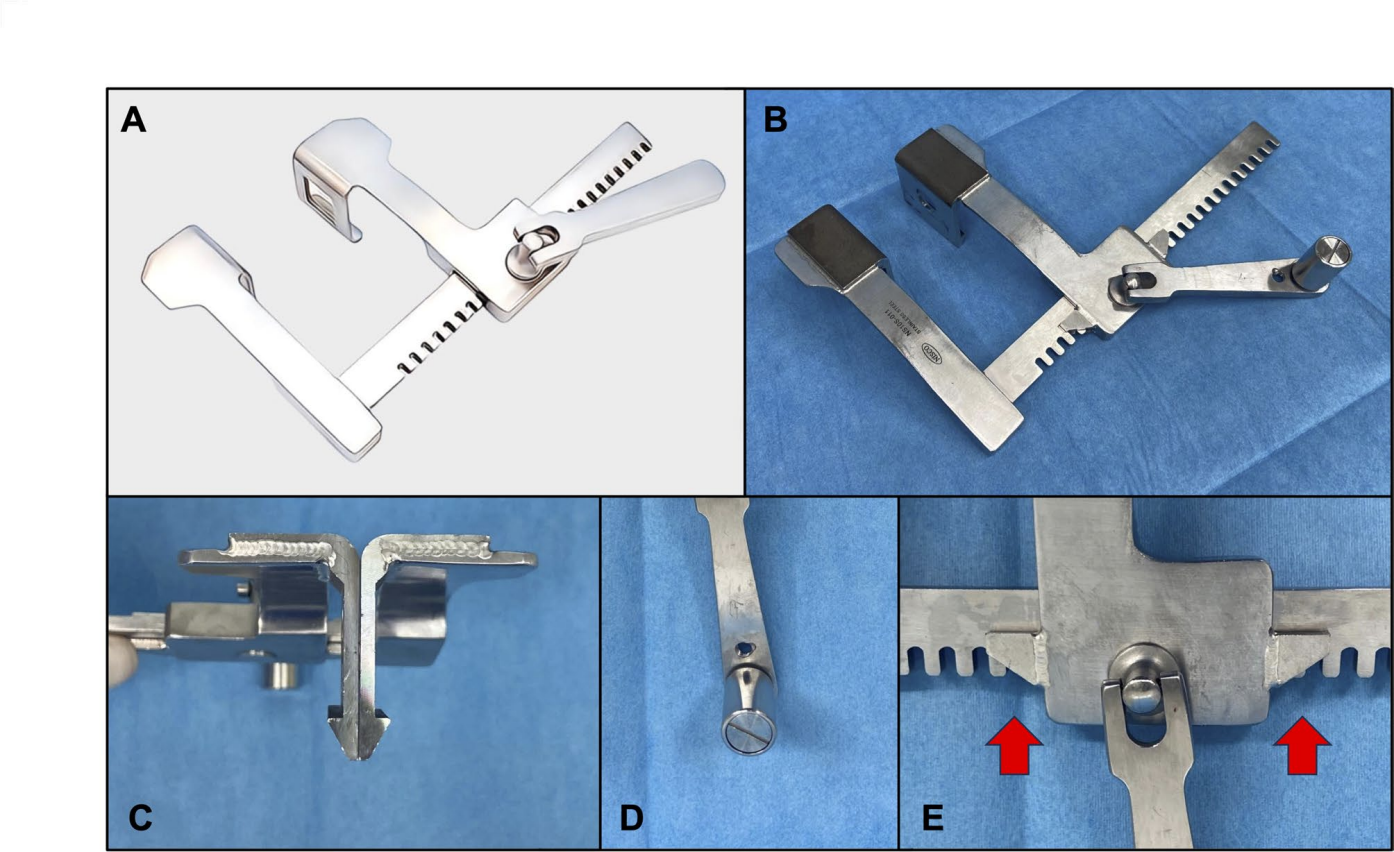
Development of the New Thoracic Retractor. **A**, Conventional finochietto retractor. **B**, Design of the new retractor. **C**, Arrow-shaped hook. **D**, Rotatable handle. **E**, Gear guard

Resuscitative thoracotomy (RT) is a critical intervention for patients in extremis due to traumatic cardiac arrest, hemorrhagic shock, cardiac tamponade, myocardial injury, or tension pneumothorax [1, 2]. Survival rates for RT vary depending on the mechanism of injury, with overall survival ranging from 5 to 15%, while blunt trauma cases have survival rates as low as 1–2% [3]. Studies have demonstrated a time-dependent relationship between RT and survival outcomes, emphasizing that thoracotomy must be performed within 10 min, as survival rates decline dramatically beyond this window, with essentially no survivors past 15 min [4, 5]. Despite the urgent nature of RT, the procedure is still performed using traditional thoracic retractors designed for scheduled surgeries (Fig. 1A). These conventional retractors present notable challenges, including difficulty in insertion within narrow intercostal spaces, excessive force requirements for operation, and frequent entrapment of surgical gloves or clothing. To address these limitations, we developed a novel thoracic retractor designed specifically for RT and evaluated its performance compared to conventional models.

## Materials and methods

### Design of the novel thoracic Retractor

The prototype of the novel thoracic retractor was developed based on the conventional finochietto retractor (NS10S-011, Nisco Co., Ltd, Tokyo, Japan) (Fig. 1B). Several key modifications were implemented: the hook shape was changed to an arrow-like design (Fig. 1C), a component was attached to the handle to enable continuously rotation (Fig. 1D), and gear guards were added to prevent surgical gloves or fabric from becoming caught in the gear mechanism during thoracic retraction (Fig. 1E).

### Experimental setup

**Fig. 2:**
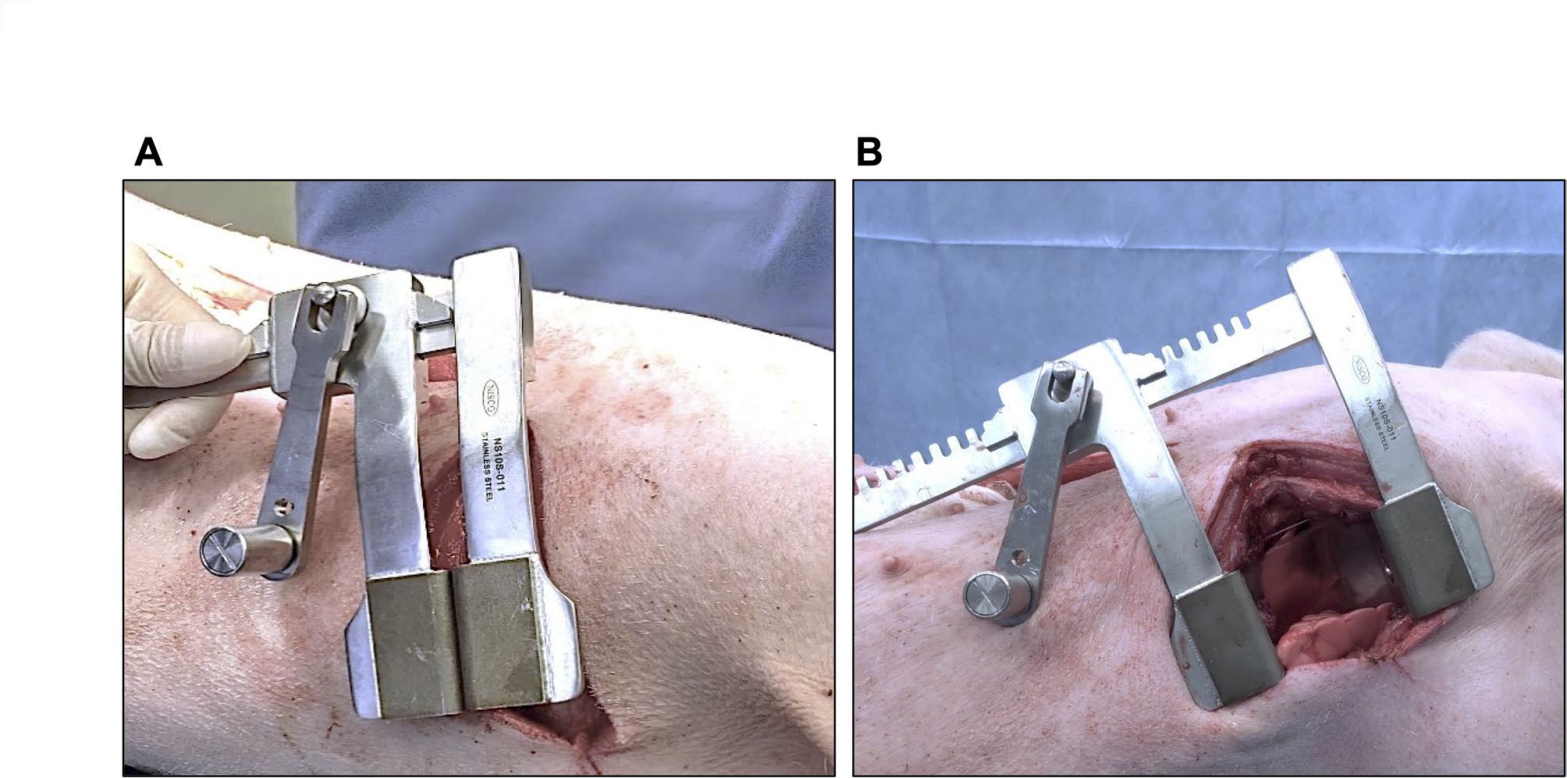
Handle Rotation Time Trial. **A**, Before rotation. **B**, After three rotations

We conducted a comparative evaluation of the performance of the novel and conventional thoracic retractors using excised porcine thoraxes. Prior to the experiment, 6 cm incisions were made in the intercostal spaces on both sides of the thorax. The evaluators inserted the retractor into the intercostal space (Fig. 2A) and rotated the handle three times (Fig. 2B). They then performed the same procedure on the opposite side of the same intercostal level using the other thoracic retractor. The orientation of the retractor (i.e., anterior or posterior handle position) was left to the discretion of each evaluator.

### Outcomes

The primary outcome was the time required (seconds) to complete three handle rotations. Secondary outcomes included ease of insertion, ease of rotation, and hook stability, each rated on a 6-point scale (1 = very difficult/unstable, 6 = very easy/stable).

### Statistical analysis

All data were expressed as median (interquartile range, IQR). Comparisons between the novel and conventional retractors were conducted using Wilcoxon signed-rank tests. A *p* value < 0.05 was considered to indicate statistical significance. All Statistical analyses were performed using a dedicated statistical software package (JMP Pro, version 17.2.0, SAS Institute, Inc., Cary, NC, USA) on a universal personal computer.

## Results

### Time required for three handle rotations

**Fig. 3:**
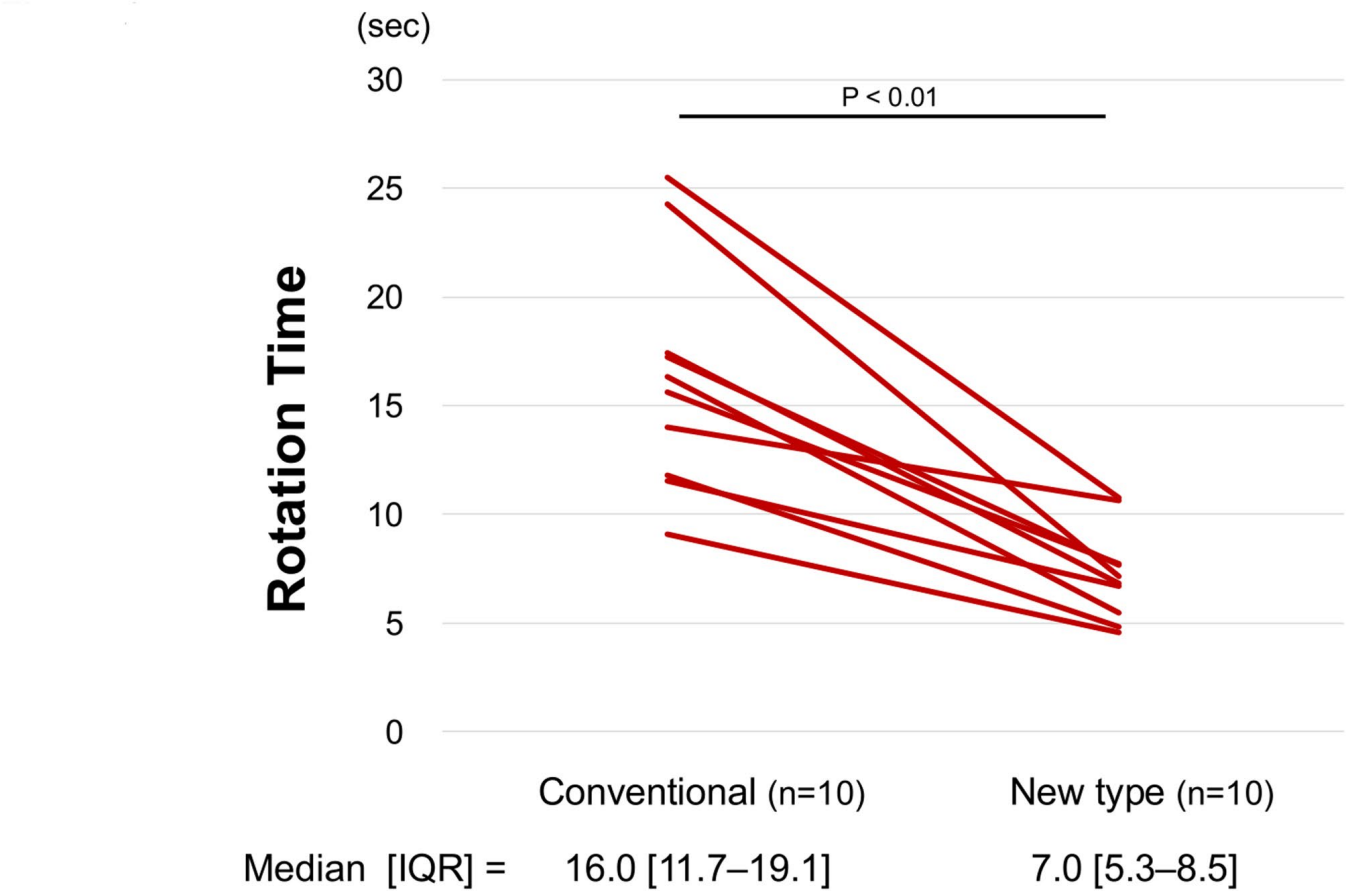
Result of Rotation Time

Thoracotomy was performed on excised porcine thoraxes using both the novel and conventional retractors by 10 surgeons, comprising 8 emergency surgeons and 2 gastrointestinal surgeons. Each surgeon performed the procedure once with each retractor, resulting in a total of 20 thoracotomy procedures. The time required to complete three handle rotations was significantly shorter with the novel retractor compared to the conventional model (16.0 [11.7–19.1] sec vs. 7.0 [5.3–8.5] sec, *P* < 0.01) (Fig. 3). All evaluators recorded shorter procedural times with the novel retractor, and no instances were observed in which the conventional retractor outperformed the novel model in terms of time required for rotation.

### Ease of insertion

**Fig. 4:**
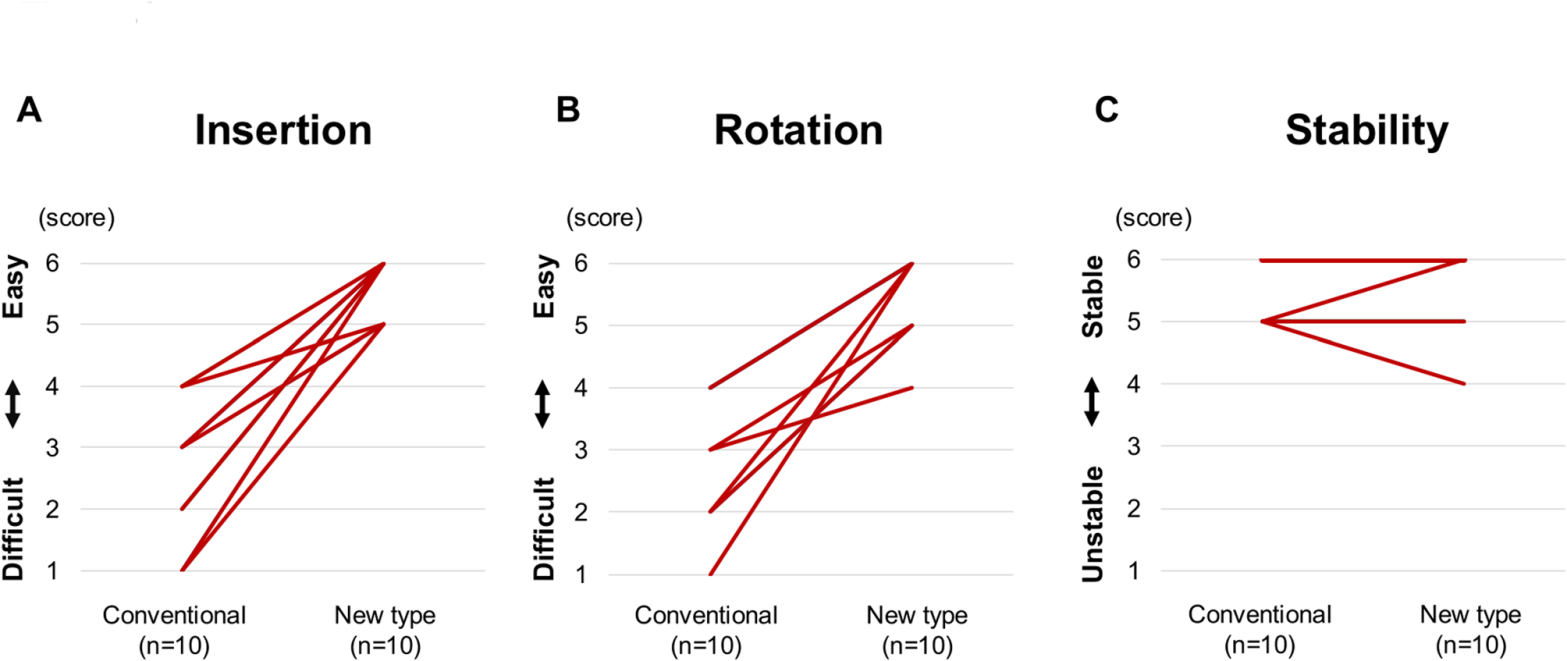
Evaluations of Functional Performance. **A**, Ease of Insertion. **B**, Ease of rotation. **C**, Hook stability

The ease of insertion was rated significantly higher for the novel retractor compared to the conventional model (6.0 [5.5–6.0] vs. 2.5 [2.0–3.0], *P* < 0.01) (Fig. 4A). The ratings were consistent across all evaluators, with minimal variability in scoring for the novel retractor. In contrast, the conventional retractor exhibited wider score dispersion, suggesting greater variability in insertion difficulty among different trials. No evaluator rated the novel retractor’s insertion ease below 5, whereas scores for the conventional retractor ranged from 2.0 to 3.0.

### Ease of rotation

The ease of rotation was also rated significantly higher for the novel retractor (5.5 [5.0–6.0] vs. 2.5 [1.0–3.5], *P* < 0.01) (Fig. 4B). The novel retractor consistently received higher scores, with all evaluators rating it at 5.0 or above, whereas the conventional retractor displayed a broader range of lower ratings, with some evaluators rating it as low as 1.0.

### Hook stability

There was no significant difference in hook stability between the novel and conventional retractors (5.0 [5.0–6.0] vs. 5.0 [5.0–5.8], *P* = 1.0) (Fig. 4C). The hook of both retractors maintained stable engagement within the intercostal space throughout the trials.

## Discussion

RT is a critical intervention, primarily performed for patients in traumatic cardiac arrest or hemorrhagic shock [1, 2]. Given the urgency of RT, procedural efficiency is critical, as survival rates decline significantly if thoracotomy is not performed within the golden 10 min [4–6]. Current guidelines, including the Denver Emergency Department Thoracotomy Guidelines and Advanced Trauma Life Support Guidelines emphasize that RT is most effective when performed within 10–15 min of cardiac arrest, particularly in penetrating trauma cases where signs of life (SOL) are present [7–9]. The Eastern Association for the Surgery of Trauma guidelines further support these indications, stressing that blunt trauma cases should only be considered for RT if cardiac arrest has occurred within 5 min and SOL is evident [10]. Although the therapeutic effectiveness of RT is closely linked to time efficiency, traditional thoracic retractors, designed for scheduled surgeries, are still used in today’s medical practice. In many real-world trauma environments, RT must often be initiated under suboptimal conditions [11, 12]. The procedure may begin before clothing is fully removed, increasing the risk of glove or fabric entrapment in the retractor’s gear mechanism. Additionally, conventional retractors are designed primarily to ensure stability during scheduled operations and are not optimized for rapid insertion or efficient thoracic expansion. This often results in extended time required for intercostal placement and chest opening, which is a disadvantage in RT where every second is critical.

In recent years, Resuscitative Endovascular Balloon Occlusion of the Aorta (REBOA) has gained significant attention as a minimally invasive alternative technique in trauma management. As it has been reported to improve short-term hemodynamic stability and reduce mortality in selected patients, many trauma centers have integrated it into their protocols [13–15]. However, its indications remain limited to specific situations, such as non-cardiac hemorrhage, and it also requires imaging guidance for accurate placement, which may not always be feasible in emergency trauma settings [16, 17]. In addition, RT provides more rapid access to aortic occlusion than REBOA, which may be delayed when arterial access is difficult [18, 19]. Consequently, in cases involving pericardial tamponade or direct cardiac injury that require immediate thoracic access, RT remains an indispensable procedure [20–22]. 

To address these limitations, we developed a novel thoracic retractor specifically designed for RT, incorporating three key modifications: a continuously rotatable handle, an arrow-shaped hook for easier intercostal insertion, and gear guards to prevent the entrapment of gloves or clothing. The continuously rotatable handle eliminates the need for the operator to reposition their grip during thoracotomy, enabling a smoother and faster opening process. Our findings confirmed that the novel retractor had a shorter rotation time and allowed for easier rotation, suggesting a potential clinical advantage in RT. Although placing the retractor handle posteriorly is generally standardized during RT, in some case, we indicate intentional anterior placement of the retractor handle depending on surgical positioning and access strategy. In current study, we allowed evaluators to determine the orientation based on their preference in this study. While this could pose a risk regarding consistency in device handling, none of the evaluators changed the handle orientation between the novel and conventional retractors. Therefore, we believe that the paired comparisons were conducted under equivalent conditions. The arrow-shaped hook was designed to facilitate insertion even in narrow intercostal spaces, which are often challenging with conventional retractors. Ease of insertion was rated significantly higher with the novel retractor, indicating that the hook modification effectively improved maneuverability. Furthermore, the hook stability assessment showed no significant difference between the novel and conventional retractors, suggesting that while the novel design facilitated easier insertion, it did not compromise the ability to maintain a secure hold within the intercostal space. Meanwhile, it is important to consider how the anatomical differences between porcine and human thoraxes may influence the translation of these findings to clinical settings. Compared to humans, pigs have a more flattened thoracic shape, narrower and more steeply angled intercostal spaces, and reduced chest wall compliance [23]. Due to these anatomical differences, thoracotomy procedures in humans are generally considered to be less technically demanding than in pigs. Therefore, the performance of the novel retractor observed in the porcine model is expected to translate into clinical practice.

Although no quantitative data are available, our practical experience suggests that difficulty in inserting the retractor into the intercostal space can lead to the most critical time loss during resuscitative thoracotomy. Therefore, ease of intercostal insertion was one of the most carefully prioritized aspects in the design of the novel retractor. At the developing phase, we prototyped two different hook shapes—an arrow-shaped tip and a serrated-tip design (Supplementary Figure). Although the serrated hook allowed for easier insertion, it occasionally failed to maintain stable position and became dislodged during chest opening. Based on these findings, the arrow-shaped hook was selected as it provided a more balanced performance in terms of both ease of insertion and stability. These findings demonstrate that the novel thoracic retractor offers improved ease of insertion compared to the conventional model while maintaining equivalent stability during thoracic retraction. While the arrow shape might appear more invasive, it has a blunt tip, ensuring that thoracic organs are not at risk of injury. Compared to conventional retractors, this design may be considered more invasive during insertion into intercostal engagement. However, considering that time is the most critical factor in RT survival, we prioritized ease of insertion over minimizing invasiveness to enhance procedural efficiency. Additionally, gear guards were implemented to prevent the entrapment of surgical gloves or clothing, a common issue with conventional retractors that can disrupt the procedure. Many emergency surgeons may have encountered this complication during RT. This problem tends to occur more frequently in disorganized or chaotic environments and can make the situation even worse, making it a critical concern. However, although a suitable experimental setup to quantitatively evaluate the effectiveness of the gear guards could not be established in this study, our hands-on experience with the novel retractor gave us a strong impression of their utility. We believe that their benefit should be further evaluated in real-world clinical settings.

This study has several limitations. First, although porcine thoraxes were appropriate for this first-stage evaluation, they do not fully replicate the anatomical and situational challenges of emergency RT in humans, such as chest wall compliance and skin resistance. Second, the assessments of ease of insertion, rotation, and hook stability were based on subjective evaluator ratings, which may have been influenced by prior familiarity or expectations. While blinding was not feasible due to the visible differences in device design, we attempted to minimize bias by providing standardized instructions and randomizing the order of device use. Third, we did not assess the degree of tissue trauma associated with different hook shapes. Finally, as the effectiveness of the gear guards could not be quantitatively assessed in this model, further research is needed to evaluate their clinical utility.

## Conclusion

We developed a novel thoracic retractor optimized for RT. Compared to conventional retractors, our new thoracic retractor enables easier intercostal insertion and faster thoracotomy. Our retractor is highly suitable for RT, where time is strongly associated with patient prognosis.

## Electronic supplementary material

Below is the link to the electronic supplementary material.

Supplementary Material 1

**Supplementary Material 2**: **Supplementary Video 1**: Usability of conventional retractor in porcine intercostal thoracotomy.

**Supplementary Material 3**: **Supplementary Video 2**: Usability of new retractor in porcine intercostal thoracotomy.

## Data Availability

Data sets generated during the current study are available from the corresponding author on reasonable request.
