# Supplementary figures and images for: Development of novel thoracic retractor for resuscitative thoracotomy
Source: Scand J Trauma Resusc Emerg Med. 2025 Jun 17;33:106. doi: 10.1186/s13049-025-01423-1 (PMC12172248; doi:10.1186/s13049-025-01423-1)

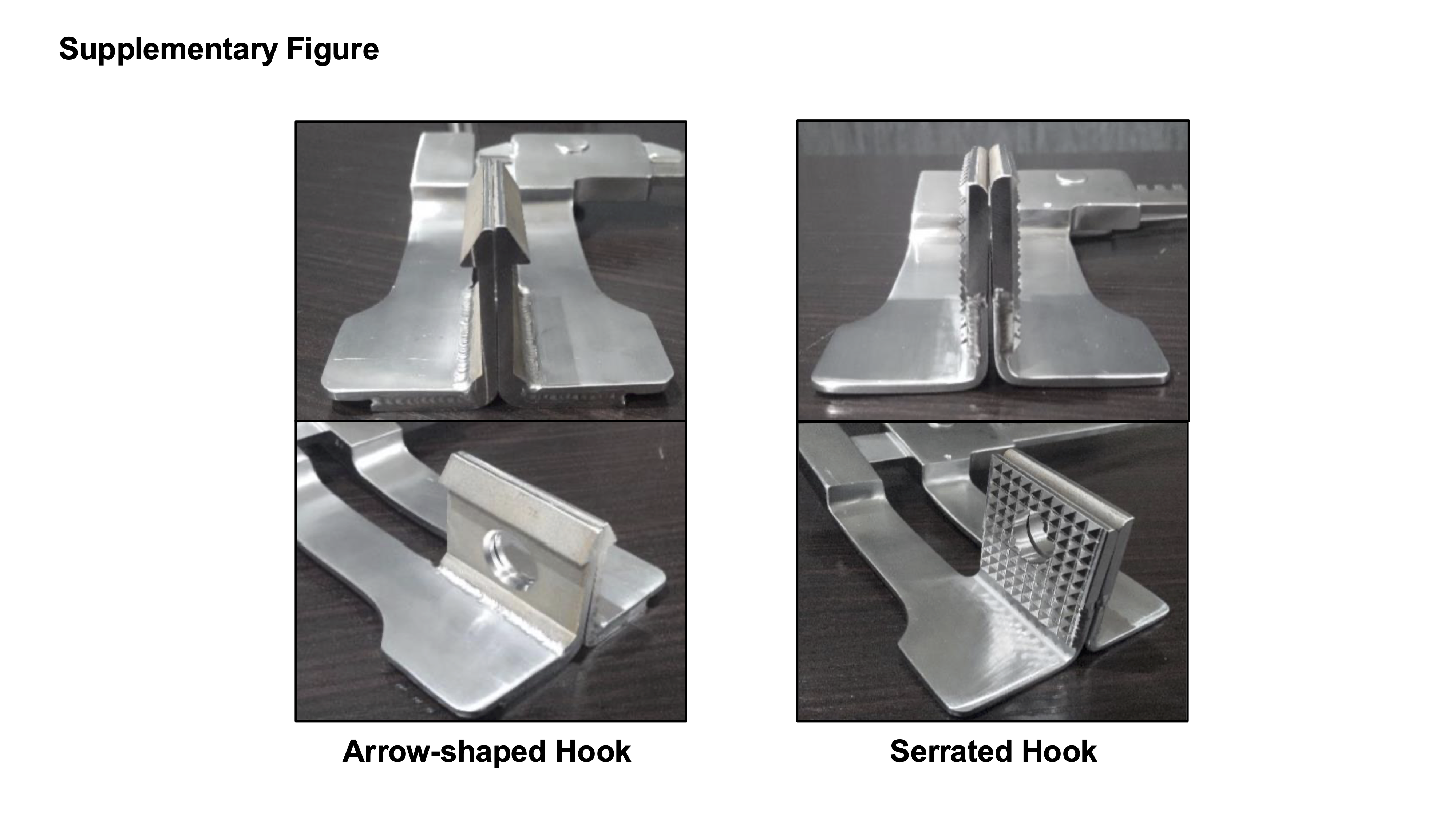

Supplement: Supplementary file 1 — Supplementary Material 1 [file 13049_2025_1423_MOESM1_ESM.tiff]
